# Association of Anti-tissue Transglutaminase Antibody Titers and Duodenal Biopsy Findings in Pediatric Patients of Celiac Disease

**DOI:** 10.7759/cureus.13679

**Published:** 2021-03-03

**Authors:** Kanchan Taneja, Nidhi Mahajan, Anuradha Rai, Sonali Malik, Arti Khatri

**Affiliations:** 1 Department of Biochemistry, Chacha Nehru Bal Chikitsalaya, Delhi, IND; 2 Department of Pathology, Chacha Nehru Bal Chikitsalaya, Delhi, IND; 3 Department of Pediatrics, Chacha Nehru Bal Chikitsalaya, Delhi, IND; 4 Department of Pathology, Gajara Raja Medical College, Gwalior, IND

**Keywords:** celiac disease/complication, serology testing, histopathology (hp), giardiasis

## Abstract

Aims & Objectives

To ascertain the association of serum anti-tissue transglutaminase (anti-tTG) antibody titers with the severity of duodenal mucosal damage on histology andto predict a possible cut-off value of anti-tTG antibody titers for the diagnosis of Celiac disease. Marsh grading greater than two in conjunction with clinical assessment, which may help avert an invasive endoscopic procedure, especially in medically unfit children.

Materials & Methods

A retrospective study was designed wherein demographic and laboratory data of children aged less than 12 years with raised anti-tTG antibody titers with available histopathology of duodenal biopsies were extracted from the hospital medical records and reviewed.

Results

A total of 134 children were included in the study, which showed female preponderance. Histopathological changes, characteristic of Celiac disease, were observed in 116 cases; seven among the rest showed evidence of Giardiasis, and 13 could be considered potential Celiac. Of the 116 patients, 1.7% belonged to Marsh grade I, 5.2% grade II and 8.6%, 26.7%, and 57.7% to grade IIIA, IIIB, and IIIC, respectively. A significant association was found between anti-tTG antibody titers and Marsh grading. The cut-off value of anti-tTG antibody titer levels for diagnosing Celiac disease using receiver operating characteristics (ROC) curve in predicting Marsh greater than two at histology was observed to be 84.6 U/ml with sensitivity, specificity, positive predictive value (PPV), and negative predictive value (NPV) of 91.7%, 68.4%, 94.2%, and 59%, respectively.

Conclusion

An anti-tTG antibody titer greater than 10 times the upper limit of normal (≥84 U/ml) is significantly associated with Marsh grade greater than two. Standard stool microscopy may be used as a simple tool in the workup of all children with raised anti-tTG antibody levels to rule out Giardiasis to avert unnecessary endoscopic evaluation for Celiac disease in such cases.

## Introduction

Celiac disease (CD) is a chronic immune-mediated enteropathy of the small intestine precipitated due to an expo­sure to dietary gluten in genetically susceptible individuals of all ages [1]. Clinical symptoms may vary from classical features, such as diarrhea, muscle wasting, failure to thrive, weight loss, poor appetite, steatorrhea, to non-classical features, such as anemia, angular stomatitis, skeletal abnormalities, and liver dysfunctions [2]. Epidemiological studies have shown that CD is one of the most common genetic diseases in the world population. This disease prevalence in a pediatric age group is around one percent worldwide [3,4].

Screening for CD is usually done by serologic testing, which includes the estimation of Celiac specific antibodies as anti-endomysial antibodies (EMAs), anti-tissue transglutaminase (anti-tTG) antibodies and anti-deamidated gliadin derived peptides. Anti-tTG antibody titers are the most preferred initial screening test for the CD because of high sensitivity and specificity [5,6]. However, a confirmatory diagnosis of CD is based on duodenal biopsy demonstrating histological features such as intraepi­thelial lymphocytosis, crypt hyperplasia, and vary­ing degrees of villous atrophy, graded according to modified Marsh classification (Marsh grade I to IIIC) [7].

Recently, the clinical practice of performing duodenal biopsies in all patients with raised anti-tTG antibody titers has been debated, and a hypothesis has been put forth that a cost-effective, reliable diagnosis of CD could be made without duodenal biopsy in patients with high titers of the same. This is specifically important in the pediatric age group where pediatric endoscopy facilities may not be available in all hospital setups and for children unfit for such invasive procedures [8-10]. European Society of Pediatric Gastroenterology, Hepatology and Nutrition (ESPGHAN) guidelines published in 2012 have suggested that the diagnosis of CD can be established without small intestine biopsy in genetically predisposed children who are symptomatic and have anti-tTG antibody titers 10 times greater than the upper limit of normal (ULN), positive EMA and good response to a gluten-free diet (GFD) [11]. However, minimal data from India are available primarily in the pediatric age group for ascertaining the association of serum anti-tTG antibody levels with duodenal histological damage and whether it has a substantial positive predictive value (PPV) to be exclusively used for the diagnosis of CD [12].

The study was therefore aimed to assess the association of anti-tTG antibody titers with the histological changes in duodenal biopsies. Also, to establish cut-off values of anti-tTG with respect to modified Marsh grading greater than two.

## Materials and methods

The present study was carried out at a tertiary pediatric care center in northern India. Clinical and laboratory records were reviewed retrospectively from January 2016 to January 2020. The data of 136 patients evaluated for CD for any reason with complete workup (high anti-tTG antibody titers, endoscopic evaluation, and histopathology) were analyzed. The cases with negative serology were excluded from the study.

 Anti-tTG antibody titers had been tested for all patients in the clinical chemistry laboratory using an enzyme-linked immunosorbent assay (ELISA) by a commercially available kit (antibody levels above 8 U/ml were considered positive, as per the manufacturer's recommendation).

Upper gastrointestinal (GI) endoscopy under conscious sedation had been performed for all patients. A minimum of four duodenal biopsies was taken, the specimen was oriented, and stained with hematoxylin and eosin. All biopsies were then reviewed and reported by the three expert pathologists of the same hospital. The histopathological severity was documented as per the modified Marsh classification, i.e., Grade 0 - normal; Grade I - increased intraepithelial lymphocytes; Grade II - increased intraepithelial lymphocytes and crypt hyperplasia; Grade IIIA - partial villous atrophy; Grade IIIB- subtotal villous atrophy; and Grade IIIC - complete villous atrophy.

Demographic data, clinical symptoms, and medical history of all the patients were extracted from hospital records. Laboratory data, including com­plete blood count (CBC) and thyroid func­tion tests of the patients available, were documented.

Statistical analysis was done using computer software (SPSS for Windows, Version 20.0; SPSS Inc., Chicago, IL, USA). Quantitative values are expressed as means and standard deviations (SD), and qualitative values are expressed in percentages and proportions. The Student's t-test for independent samples was used to compare the mean values of continuous variables. An analysis of variance (ANOVA) test was used to observe an association between Marsh grading and anti-tTG antibody titers. The Tukey-Kramer post-hoc test was performed to compare the mean of each Marsh grading. The receiver operating characteristics (ROC) curve was used to determine the anti-tTG antibody titer cut-off for best sensitivity, specificity, PPV, and negative predictive value (NPV) with a 95% confidence interval (CI) and 0.05 alpha error. Pearson's coefficient correlation analysis was done to assess the association of hemoglobin with anti-tTG antibody titers. A p-value of less than 0.05 was considered statistically significant.

## Results

A total of 134 children were included in this study, of which predominantly 61.2% (82) were females. The mean age at diagnosis was 7.49 ± 3.03 years. The mean age of males and females was similar (7.35±3.17 years and 7.59±2.94 years, respectively). The demographic and clinical characteristics of this study are given below (Table 1).

**Table 1:** Demographic and clinical characteristics of the patients

Demographic characteristics	Number (Total number of patients)	Percentage of total (%)
Age		
≤5 years	40(134)	29.9
>5 years	94(134)	70.1
Sex		
Male	52(134)	38.8
Female	82(134)	61.2
Gastrointestinal symptoms		
Chronic diarrhea	88	65.7
Abdominal pain	24	17.9
Vomiting	07	5.2
Abdominal distention	07	5.2
Non-gastrointestinal manifestations		
Weight loss/not gaining weight	26	19.4
Anemia/pallor	20	14.9
Rickets	01	0.7
Short stature	11	8.2

The most common GI symptom was chronic diarrhea observed in 88 (65.7%), followed by abdominal pain in 24 (17.9%), vomiting in 7 (5.2%), and abdominal distention in 7 (5.2%) patients. Non-GI symptoms like weight loss or not able to gain weight were observed in 26 (19.4%), anemia in 20 (14.9%), and short stature in 11 (8.2%) patients. One patient had diabetes mellitus, and one was diagnosed to have rickets. We had records of the thyroid profile of 68 patients, and none of them was observed to have any thyroid-related abnormality.

Regarding the histopathological changes, of the 134 patients, 116 (86.5%) were observed to have histopathological changes, and 18 did not have any histological findings pertaining to the CD. However, among these 18 patients, 7 (5.2%) were observed to have Giardia trophozoites adhered to the intestinal mucosa, and the rest 11 (8.2%) could be considered potential Celiac. Of the 116 patients with histopathological changes 2 (1.7%) belonged to Marsh grade I, 6 (5.2%) were Marsh grade II, and 10 (8.6%), 31 (26.7%), and 67 (57.7%) belonged to grade IIIA, IIIB, and IIIC, respectively.

The assessment of the association of anti-tTG antibody titers with the histopathological changes of duodenal biopsies is given in Table 2.

**Table 2:** Marsh grading and anti-tTG antibody titers anti-tTG: anti-tissue transglutaminase

Marsh Grading	Mean anti-tTG antibody titers (U/ml)	Number	<80 U/ml	>80 U/ml	<120 U/ml	>120 U/ml
0 & I	57.45±48.57	13	12	1	12	1
II	125.02±96.03	6	3	3	6	0
III A	151.25±113.72	10	6	6	6	6
III B	186.61±100.33	31	11	20	16	15
III C	254.04±103.52	67	7	58	17	48
0 with Giardiasis	162.89±146.71	7	4	3	4	3

Out of 127 patients (excluding cases with Giardiasis), 107 patients (93.4%) had anti-tTG antibody titers ≥80 U/ml, of which 100 had Marsh grade III, 5 patients (4.6%) had Marsh grade I or Marsh grade II, and only 2 patients (1.9%) had no histological changes (Marsh 0). Among 20 patients with anti-tTG antibody titers <80 U/ml, 9 (45%) had normal duodenal biopsies (potential CD), while 11 of them (55%) had histological changes suggestive of CD. Further analysis to assess the association between the anti-tTG antibody titers and Marsh grading was done and found to be significant (Table 3).

**Table 3:** Association of Marsh grading with anti-tTG antibody titers

Marsh grading	Mean anti-tTG antibody titers (U/ml)	Number	P-value
0 & I	57.45±48.57	13	<0.001
II	125.02±96.03	6	
III A	151.25±113.72	10	
III B	186.61±100.33	31	
III C	254.04±103.52	67	

Post-hoc tests were performed and showed that the mean anti-tTG antibody titers differed significantly between grades II and III C, III A and III C, III B and III C.

**Figure 1:**
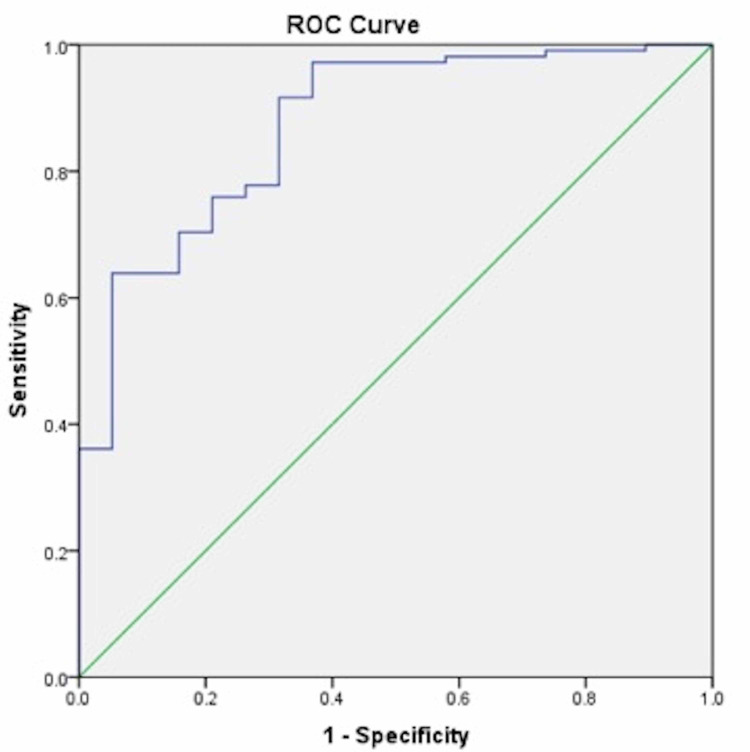
Receiver operating characteristic (ROC) curve showing maximum area under curve for Marsh grade more than II in biopsy suggestive of celiac disease

ROC curve analysis showed an area under the curve (AUC) of 0.87 [with the standard error of 0.043 and 95% confidence interval (0.79 to 0.96)] and while exploring the accuracy of anti-tTG antibody titers for diagnosing CD using ROC curve the best cut-off value of anti-tTG antibody titers in predicting Marsh grade more than II at histology was observed to be 84.6 U/ml (almost 10 times the upper normal value), with sensitivity, specificity, PPV and NPV of 91.7%, 68.4%, 94.2%, and 59%, respectively (Figure 1).

It was observed that patients with biopsy suggestive of CD (Marsh grading ≥ II) had lower mean hemoglobin as compared to patients with negative biopsy (Marsh 0 & I grading) (8.41±1.38 g/dl vs. 9.53±2.01 g/dl), which was near to significant statistically (Table 4).

**Table 4:** Association between Marsh grading and hemoglobin levels

Marsh grading	Hemoglobin levels (g/dl)	P-value
0 & I	9.53±2.01	0.052
≥ II	8.41±1.38	

Similarly, a significant inverse correlation was also observed between hemoglobin and rising anti-tTG antibody titers using Pearson's coefficient correlation (Table 5).

**Table 5:** Correlation between hemoglobin levels and anti-tTG antibody titers

Parameter	Parameter	Pearson’s correlation
Hemoglobin levels (g/dl)	Anti-tTG antibody titers (U/ml)	p=0.025 (r=0.199)

## Discussion

Diagnosis of CD relies upon clinical, serological, genetic, and histopathological parameters. Of these, duodenal histopathology is considered the gold standard. However, it has its own limitations. Clinical disorders that mimic similar microscopic picture, invasiveness, high-cost logistics, false positivity due to improper sample processing, sample inadequacy are some of the disadvantages that are to be debated while considering a biopsy for the diagnosis of CD, especially in a pediatric setup [13-15]. In recent years, specific antibody tests are the initial tools that are being used as a screening test to identify individuals with CD. Before using an anti-tTG antibody, antigliadin antibody, and EMA were the serologic tests considered for screening and as diagnostic tools for CD. EMA has very high specificity but low sensitivity, which makes it an unreliable tool in clinical practice for screening patients with CD [16]. Anti-tTG antibody titers were rec­ognized as the major endomysial autoantigen by Dieterich and colleagues in 1997 and have been extensively used as a sensitive and specific ELISA-based diagnosing CD [17]. Systematic reviews comparing the EMA and anti-tTG antibody concluded that the human recombinant anti-tTG antibody is the preferred test for screening asymptomatic people and for excluding disease in symptomatic individuals [18]. A meta-analysis of studies was published in recent years where the comparison between deamidated gliadin peptide antibody and anti-tTG antibody titers was made; anti-tTG antibody showed better performance and therefore remained the preferred serological test for the diagnosis or exclusion of CD [5].

Significant association of anti-tTG antibody titers with the severity of histological grading has been reported in a few of the previous studies [8,19]. Barker et al. showed that 48 of the 49 symptomatic children with anti-tTG antibody titers ≥ 100 U/ml (five times the normal cut-off) had Marsh grading of at least grade II enteropathy with a sensitivity and specificity of 98% and 97.2%, respectively [19]. Donaldson et al. showed anti-tTG antibody titers of 40 U/ml (twice the ULN) had sensitivity, specificity, PPV, and NPV of 82.1%, 98.4%, 97.9%, and 85.7%, respectively, for Marsh IIIA in 177 patients of CD in the pediatric age group [9].

The latest ESPGHAN updated guidelines for the diagnosis of CD in the pediatric age group and suggested that in symptomatic children and adolescents having anti-tTG antibody titers 10 times above the normal upper limit with confirmatory positive anti-EMA antibody and/or HLADQ2/DQ8 heterodimer, there are highly likely chances of villous atrophy (Marsh III) and can obviate the necessity of doing a histological evaluation for the diagnosis of CD [20]. Post these guidelines, studies were done to validate the guidelines.

Allesio et al. concluded that in patients of all age groups with positive anti-tTG serology ≥ seven times the cut-off along with positive EMA, the probability of duodenal damage is very high, and under specific conditions, a duodenal biopsy could be avoided in such cases for the diagnosis of CD [21]. Zanini et al. concluded that anti-tTG antibody titers more than five times the upper limit of the normal level are 100% specific for duodenal atrophy and can be used as a cut-off, which could help avoid biopsy in one-third of adult patients with CD [22]. Another study by Hawamdeh et al. showed that anti-tTg antibody levels of more than 10 times ULN correlated significantly with CD's histopathological changes. A significant correlation was reported between high anti-tTg antibody titers and higher Marsh grade [23].

Our study showed that there was a significant association between the severity of histopathological changes and high antibody titers. Patients with high antibody titers (more than 10 times the ULN) had more severe histological severity (Marsh grade II/III) compared to those with low antibody titers (Marsh grade I). The best cut-off value of anti-tTG antibody titers in predicting Marsh greater than two at histology using ROC curve was observed to be 84.6 U/ml (almost 10 times the upper normal value), with sensitivity, specificity, PPV, and NPV of 91.7%, 68.4%, 94.2%, and 59%, respectively.

Although a significant association between high anti-tTG antibody titer and severity of duodenal histology was observed in our study, seven of our patients (5.5%) having Marsh III grading on histology had their anti-tTG antibody titers less than 10 times ULN. A variable amount of gluten intake in the diet or the patient's variable immunological response could be an explanation for the above-said observation in our study. It gives an insight into the importance of assessing gluten intake in nutritional history while interpreting the anti-tTG antibody titers. Similar findings were also observed in a study by Hawamdeh et al. where 18% of the patients with Marsh grading III had anti-tTG antibody titers less than 10 times the UNL [23]. Lewis Scott also reported that 5%-16% of Celiac cases confirmed histologically had negative anti-tTG antibody titers [5].

Three (20%) of our patients with Marsh 0 had anti-tTG antibody titers more than 10 times ULN and met the definition of potential Celiacs. A possible explanation could be that histological abnormalities in CD can be patchy [24,25]. A similar observation was reported by Freeman, which showed that 20% of the patients with anti-tTG antibody titers >100 U/mL had a normal biopsy [26].

An inverse association was observed between hemoglobin levels and Marsh grading. A statistically significant inverse correlation was also noted between hemoglobin and anti-tTG antibody titers, indicating increasing severity of anemia with progressively increasing destruction of the mucosal epithelium with increasing histological severity. Similar findings have been observed in previous studies as well [27,28].

Another interesting observation in the current study was that seven of our subjects who were found to have raised anti-tTG antibody titers had evidence of intestinal Giardiasis as per biopsy reports instead of crypt hyperplasia or villous blunting (not included during statistical evaluation for ROC curve). This fundamental observation warrants further studies to be taken up to assess the anti-tTG antibody titers in Giardiasis patients to find out the incidence of such cases with raised anti-tTG antibody titers. Consecutive stool microscopy for three days as per the standard protocols should be taken up in such cases with initially raised anti-tTG antibody titers to rule out Giardiasis so as to avoid unnecessary invasive procedures.

This study's limitations include non-evaluation of anti-EMA and HLA typing in celiac patients due to this facility's unavailability in our laboratory. Also, since it is a single-center observational study covering a small population, other variables in relation to histopathological changes and anti-tTG antibody titers like family history, the autoimmune status of the individual could not be analyzed. 

## Conclusions

This study demonstrated a significant association between anti-tTG antibody titers and the duodenal histological severity grading in pediatric patients with CD. An anti-tTG titer more than almost 10 times the ULN is significantly associated with Marsh grade greater than two; an intestinal biopsy may be avoided in such cases. Caution must be taken to exclude Giardiasis by following standard stool microscopy examination to prevent false-positive CD cases. Due to variation among the cut-offs between various anti-tTG antibody measuring kits, as seen in numerous studies, assigning any universal cut-off value or multiples of UNL for diagnosing CD based on a single kit is not recommendable. A duodenal biopsy may be reserved for cases with high clinical suspicion, regardless of serological test results.
